# Analysis of MSX1, RYK, NFκB p65, and CCL4 Proteins and *MSX2*, *RYK*, and *PTX3* Genes in Human Cleft Lip Tissue

**DOI:** 10.3390/ijms262110599

**Published:** 2025-10-30

**Authors:** Mārtiņš Vaivads, Alise Elizabete Rone, Māra Pilmane

**Affiliations:** Institute of Anatomy and Anthropology, Rīga Stradiņš University, Krovalda Boulevard 9, LV-1010 Riga, Latvia; aliseelizabete.rone@rsu.lv (A.E.R.); mara.pilmane@rsu.lv (M.P.)

**Keywords:** MSX1, MSX2, RYK, NFκB p65, CCL4, PTX3, cleft lip, IHC, CISH

## Abstract

Human cleft lip morphopathogenesis is a complicated process involving multiple genes and proteins. Certain factors like muscle segment homeobox 1 (MSX1) and 2 (MSX2) as well as receptor-like tyrosine kinase (RYK) are important during lip embryogenesis, while others like nuclear factor kappa-B protein 65 (NFκB p65), C-C motif chemokine ligand 4 (CCL4), and pentraxin 3 (PTX3) regulate local inflammation and immunomodulation. The exact role of these factors in human cleft morphopathogenesis remains uncertain and limits the opportunity to improve cleft treatment and possible prophylaxis. Immunohistochemistry (IHC) for MSX1, RYK, NFκB p65, and CCL4 proteins and chromogenic in situ hybridization (CISH) for *MSX2*, *RYK*, and *PTX3* genes were used to analyze postnatal human cleft lip tissue (15 patients) and control tissue (6 patients). The semiquantitative counting method was used to assess factor/gene-signal-containing cells. Statistical analysis was performed. IHC findings showed decreased MSX1, NFκB p65, and CCL4 proteins in cleft lip connective tissue and endothelium, while RYK protein was decreased only in cleft connective tissue. CISH showed increases in *MSX2* and *RYK* gene-signal-containing cells in cleft lip tissue while *PTX3* did not differ from controls. Multiple statistically significant correlations were calculated. The findings are discussed in detail to determine their significance in cleft lip morphopathogenesis.

## 1. Introduction

A cleft lip with or without a cleft palate is one of the most common congenital anomalies found in the human population, which affects approximately 10 million individuals worldwide [1]. A cleft lip typically forms due to failed fusion of the maxillary prominence and medial nasal fold in the developing upper lip region during the 4th–6th week of embryonic development [2]. Cleft lip variations are a unilateral cleft lip, which typically affects the left side of the face more than the right side [3], and a bilateral cleft lip, which affects both sides of the upper lip region [4]. Although this is a relatively common pathology, the exact mechanisms of cleft lip formation on the tissue level continue to remain uncertain, which impacts the ability to effectively prevent cleft formation during embryogenesis or to improve existing surgical treatment options. It is complicated to understand wound healing, local inflammation, and tissue regeneration in cleft lip patients without fully knowing the exact molecular mechanisms that caused the cleft originally.

Multiple studies have assessed the significance of specific genes, certain proteins, and signaling pathways in facial cleft pathogenesis [5,6]. Most cleft morphological research has assessed the presence in certain genes or proteins within cleft-affected tissue [7,8,9]. Some of these genes and their coded proteins regulate facial tissue growth during embryogenesis while others are involved in tissue homeostasis regulation, affecting both local immune function as well as tissue regeneration and healing after cleft-correcting surgery. Studies that analyze and compare interactions of both genes and proteins within human cleft-affected tissue by using histological methods are relatively limited, which means that understanding cleft morphopathogenesis in humans is still a difficult riddle to solve and unravel. Understanding the interactions of both genetic and proteomic factors in cleft morphopathogenesis could be essential in improving cleft treatment, ensuring tissue healing after cleft-correcting surgery, as well as assessing possibilities of cleft prophylaxis.

Certain cleft candidate genes are associated with cleft pathogenesis. For example, muscle segment homeobox 1 (*MSX1*) and 2 (*MSX2*) genes encode transcription factors that regulate orofacial region morphogenesis [10]. MSX1 and MSX2 are needed for correct cranial neural crest cell differentiation [11]. Defects in the *MSX1* gene have been associated with a cleft lip and palate [12,13]. Similarly, *MSX2* gene mutations have also been associated with orofacial clefts [14]. *MSX1* and *MSX2* genes have some functional redundancy as their nucleotide sequences are partially overlapping with each other and their expression patterns in the branchial arch region are also similar, although the *MSX1* gene has been described as encoding a more potent transcription factor than the *MSX2* gene due to differences in the N-terminal region [15,16]. Another cleft candidate gene, named the receptor-like tyrosine kinase (*RYK*) gene, encodes a receptor that is involved in the non-canonical wingless-related integration site (WNT) signaling pathway [17]. Previous studies have found that the RYK protein plays a role in excessive tissue growth and cell cycle disturbances, as seen in different types of cancer [18], while it also has regulatory functions during embryonic development. For example, RYK has anti-inflammatory properties in lung mesenchyme [19]. It also regulates neural tissue differentiation [20] and inhibits neural dendrite growth and arborization [21]. Mutations in the *RYK* gene have been associated with craniofacial clefts in humans [22], although the exact role of the *RYK* gene and protein in cleft lip morphopathogenesis remain relatively unclear.

On the other hand, there are other proteins and genes that are more involved in local tissue homeostasis regulation, mainly by providing immune and immunomodulation functions. For example, nuclear factor kappa-light-chain-enhancer of activated B cells protein 65 (NFκB p65) is involved in the regulation of inflammation, cell growth, and differentiation [23]. Increased activity of NFκB p65 has been associated with increased cell proliferation as seen in oral squamous cell carcinoma [24] as well as precancerous conditions in oral cavity epithelium [25]. Expression of NFκB p65 has been observed in inflamed colorectal tissue of mice [26] and colorectal cancer tissue in humans [27]. Oral buccal mucosa and intestinal mucosa of rats have shown increased expression NFκB p65 after intestinal ischemia–reperfusion injury due to the presence of systemic inflammation [28]. The presence and involvement of NFκB p65 within cleft lip tissue morphopathogenesis have not been well described, although disturbed oral tissue growth and inflammation are typically present in cleft-affected tissue. Another protein providing immune system regulatory functions is the C-C motif chemokine ligand 4 (CCL4), which is also known as macrophage inflammatory protein 1β (MIP-1β). This protein has been well described as an inflammatory mediator that functions as a chemoattractant of various immune cells like eosinophils [29], T-lymphocytes, and macrophages [30]. The presence of CCL4 has previously been identified in tumor tissue [31], in synovial fluid of osteoarthritis patients [32], in blood of sepsis patients [33], and in traumatic brain injuries [34]. CCL4 has also previously been identified in healthy human oral mucosa tissue [35], and increased expression of CCL4 was noted in oral squamous cell carcinoma tissue of both humans and mice, indicating its possible effects in disrupting oral cavity tissue growth [36,37]. There is limited information available on CCL4’s involvement in orofacial cleft morphopathogenesis. A gene that has important immune regulatory functions is the pentraxin 3 (*PTX3*) gene, which encodes a protein that functions as a soluble pattern recognition molecule, one which regulates inflammation, humoral immunity and tissue remodeling locally in inflamed tissue [38]. *PTX3* expression has been identified in epithelial tissue [39], connective tissue [40], and endothelium and macrophages [41]. *PTX3* gene expression has been assessed in cleft-affected tissue [42].

The role of these local inflammatory factors in orofacial tissue growth and development has been described in some studies. For example, excess nuclear factor kappa-light-chain-enhancer of activated B cells (NFκB) signaling in mice oral cavity tissue results in reduced apoptosis, which causes ectopic odontogenesis [43]. CCL4 involvement in maxillary region development has been described in *Paramisgurnus dabryanus* fish species during the formation of maxillary barbel [44]. The *PTX3* gene also regulates oral tissue growth and development by affecting human dental pulp stem cell differentiation and migration—increased *PTX3* expression stimulates human dental pulp stem cell osteogenic/odontogenic differentiation while the knockdown of *PTX3* decreased differentiation and cell migration [45]. It can be theorized that these factors might also play a role in different types of oral tissue growth disturbances like orofacial clefts. As the information about these inflammatory and immunomodulatory factors in oral tissue pathologies like clefts is rather limited, the assessment of the NFκB p65 and CCL4 proteins and *PTX3* gene in postnatal human cleft lip tissue could provide additional insight about the molecular processes and tissue homeostasis disturbances seen in human postnatal cleft lip tissue. Understanding the presence and interaction between cleft candidate genes, their coded proteins, and local tissue inflammation regulators might improve our understanding of cleft tissue morphopathogenesis and could clarify what exactly happens with these factors during disturbed oral tissue growth.

The main aim of this study is to assess the presence of MSX1, RYK, NFκB p65, and CCL4 proteins by immunohistochemistry (IHC) and *MSX2*, *RYK*, and *PTX3* gene-signal-containing cells by using chromogenic in situ hybridization (CISH) in cleft lip and control tissue by using the semiquantitative counting method. An important objective of the study is to provide a comparison of both controls and cleft lip tissue in factor/gene-signal-positive cells, which will help to determine if the evaluated proteins/genes are possibly involved in postnatal cleft lip morphopathogenesis. Another objective of this study is calculating and analyzing correlations between protein/gene-signal-containing cells in cleft lip tissue and controls, to provide further insights into the interactions between the evaluated factors and their possible role in cleft lip morphopathogenesis. MSX1, RYK, NFκB p65, and CCL4 proteins and *MSX2*, *RYK*, and *PTX3* genes were selected for the study due to the availability of necessary reagents for both IHC and CISH, due to their functional role in regulating tissue growth, regeneration, immune functions, and local inflammation, and due to the limited information available on the role and interactions of these factors in cleft morphopathogenesis.

The knowledge gained about these factors and their presence in cleft lip tissue could be beneficial in improving wound healing and preventing excessive inflammation after cleft-correcting surgery while also giving more insight into cleft tissue formation, which could be useful in developing improved cleft prophylaxis methods in the future.

## 2. Results

### 2.1. Immunohistochemistry

Immunohistochemical analysis indicated the presence of MSX1, RYK, NFκB p65, and CCL4 in all patient and control group specimens, with a variable distribution of immunoreactive cells.

**Figure 1:**
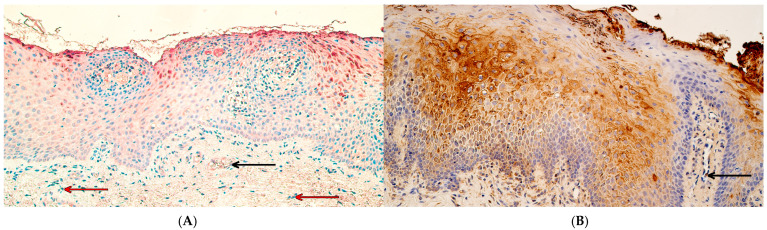
MSX1 immunohistochemistry in control and cleft lip tissue. (**A**) Control tissue with few to moderate number of MSX1-containing epithelial cells and a rare occurrence of MSX1-positive connective tissue cells (red arrows) and endotheliocytes (black arrow), MSX1 IHC, 200×. (**B**) Cleft lip tissue with moderate to numerous MSX1-containing epithelial cells, a moderate number of MSX1-positive connective tissue cells, and a few MSX1-positive endothelial cells (black arrow), MSX1 IHC, 200×.

The MSX1 protein was found in every individual within the patient and control groups, with a variable distribution. The median number of MSX1-positive cells in control tissue was as follows: moderate to numerous (++/+++) in the surface epithelium, moderate (++) in the connective tissue, and moderate (++) in the endothelium (Figure 1A). In the patient group, the median number of MSX1-positive cells was moderate (++) in the surface epithelium, a few (+) in connective tissue, and a few (+) in positive endothelial cells (Figure 1B). Statistically significant differences between controls and the patient group were noted in the number of MSX1 immunoreactive cells in the connective tissue (U = 5.0, *p* = 0.001) and endothelium (U = 2.0, *p* < 0.001) but not in the surface epithelium (U = 30.5, *p* = 0.267).

**Figure 2:**
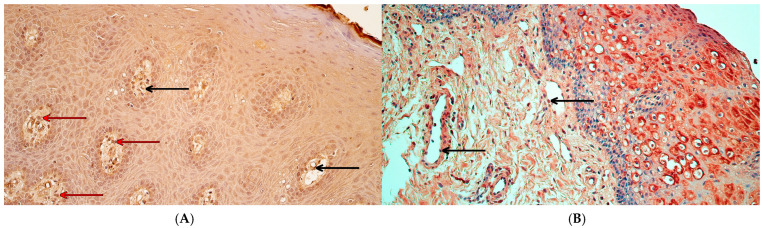
RYK immunohistochemistry in control and cleft lip tissue. (**A**) Control tissue with numerous RYK-positive epithelial cells and moderate to numerous RYK-containing connective tissue cells (red arrows) and endotheliocytes (black arrows), RYK IHC, 200×. (**B**) Cleft lip tissue with numerous RYK-containing epithelial cells and a moderate number of RYK-positive connective tissue cells and endotheliocytes (black arrows), RYK IHC, 200×.

RYK protein-positive cells were also identified in every tissue sample from both control and patient groups. The median number of RYK-positive cells in the control group was numerous (+++) positive cells in the epithelium, connective tissue, and endothelium (Figure 2A), while in the patient group, the median number of RYK-containing cells was numerous (+++) in the epithelium, moderate (++)in the connective tissue, and moderate to numerous (++/+++) in the endothelium (Figure 2B). A statistically significant difference between controls and patients in RYK immunoreactivity was identified in the connective tissue (U = 12.0, *p* = 0.008), but not in the surface epithelium (U = 33.5, *p* = 0.381) or endothelium (U = 23.5, *p* = 0.095).

**Figure 3:**
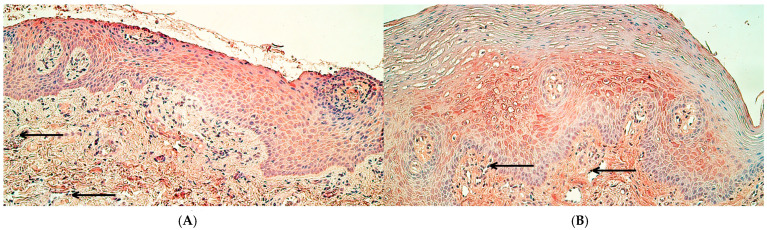
NFκB p65 immunohistochemistry in control and cleft lip tissue. (**A**) Control tissue with numerous NFκB p65-containing epithelial cells, few to moderate NFκB p65-positive connective tissue cells, and a few NFκB p65-positive endothelial cells (black arrows), NFκB p65 IHC, 200×. (**B**) Cleft lip tissue with a moderate number of NFκB p65-containing epithelial cells, few to moderate NFκB p65-containing connective tissue cells, and a few NFκB p65-positive endothelial cells (black arrows), NFκB p65 IHC, 200×.

NFκB p65 was identified in every tissue sample, with a variable distribution. In control tissue, the median number of NFκB p65-positive cells was moderate to numerous (++/+++) in the surface epithelium and moderate (++) in the connective tissue and endothelium (Figure 3A). In the patient group, the median number of NFκB p65-positive cells in the epithelium was moderate (++), and in connective tissue and endothelium, was a few (+) (Figure 3B). A statistically significant difference between controls and patients in NFκB p65 immunoreactivity was identified in the connective tissue (U = 7.0, *p* = 0.002) and endothelium (U = 2.0, *p* < 0.001) but not in the surface epithelium (U = 35.5, *p* = 0.470).

**Figure 4:**
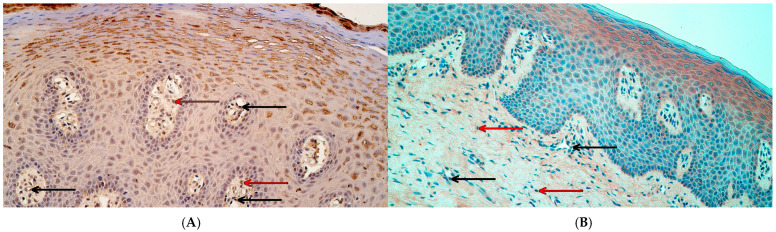
CCL4 immunohistochemistry in control and cleft lip tissue. (**A**) Control tissue with few to moderate number of CCL4-containing epithelial cells and a few CCL4-positive connective tissue cells (red arrows) and endothelial cells (black arrows), CCL4 IHC, 200×. (**B**) Cleft lip tissue with a moderate number of CCL4-positive epithelial cells and a rare occurrence of CCL4-positive connective tissue cells (red arrows) and endothelial cells (black arrows), CCL4 IHC, 200×.

CCL4-containing cells were visible in every tissue sample. The median number of CCL4-positive cells in controls was between a few to moderate and moderate (+/++–++) in the epithelium, moderate (++) in connective tissue, and few to moderate (+/++) in the endothelium (Figure 4A). In the patient group, the median number of CCL4-containing cells was moderate (++) in the surface epithelium, a few (+) in connective tissue, and a rare occurrence (0/+) in the endothelium (Figure 4B). Statistically significant differences between controls and patients in CCL4 immunoreactivity were identified—a significant difference was seen in the connective tissue (U = 10.5, *p* = 0.005) and endothelium (U = 6.0, *p* = 0.001) but not in the surface epithelium (U = 44.0, *p* = 0.970).

The median values of immunohistochemically evaluated proteins within each group are available in Table 1.

### 2.2. Chromogenic In Situ Hybridization

*MSX2*, *RYK*, and *PTX3* gene-signal-containing cells were identified in both the control and patient groups.

**Figure 5:**
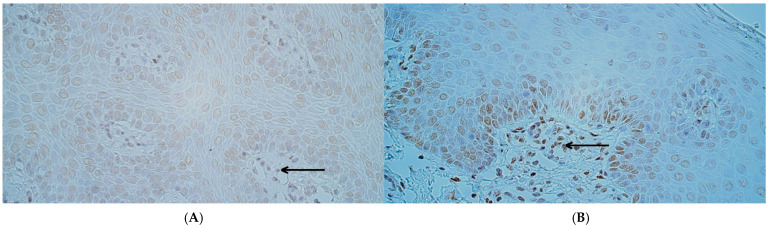
*MSX2* gene visualization in control tissue and cleft lip tissue using chromogenic in situ hybridization. (**A**) Control tissue with a moderate number of *MSX2* gene-signal-containing cells in the epithelium and connective tissue and a rare occurrence of *MSX2* gene-signal-containing endothelial cells (black arrow), *MSX2* CISH, 400×. (**B**) Cleft lip tissue with few to moderate number of *MSX2* gene-signal-containing cells in the epithelium and connective tissue cells and a few *MSX2* gene-signal-containing endothelial cells (black arrow), *MSX2* CISH, 400×.

The median values of *MSX2* gene signals in the control group were the following—a rare occurrence (0/+) in the epithelium, and between no gene-signal-containing cells and a rare occurrence (0–0/+) in the connective tissue and endothelium (Figure 5A). In the patient group, the median values of *MSX2* gene signals were a few to moderate (+/++) in the epithelium and connective tissue, while in the endothelium, they were a few (+) (Figure 5B). Statistically significant differences in the number of *MSX2* gene-signal-containing cells were identified between controls and the patient group in the surface epithelium (U = 11.5, *p* = 0.006), in connective tissue (U = 12.0, *p* = 0.008), and in the endothelium (U = 13.5, *p* = 0.011).

**Figure 6:**
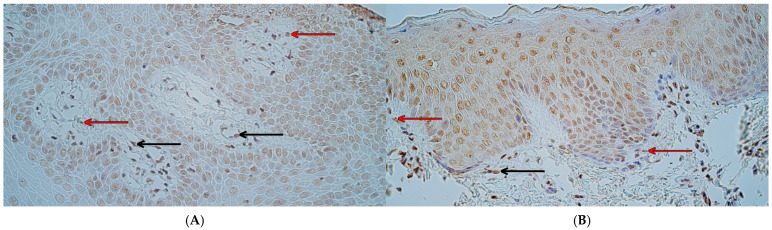
*RYK* gene visualization in control tissue and cleft lip-affected tissue using chromogenic in situ hybridization. (**A**) Control tissue with moderate to numerous *RYK* gene-signal-containing epithelial cells and a few *RYK* gene-signal-containing connective tissue cells (red arrows) and endothelial cells (black arrows), *RYK* CISH, 400×. (**B**) Cleft lip tissue with numerous *RYK* gene-signal-containing epithelial cells and a few *RYK* gene-signal-containing connective tissue cells (red arrows) and endothelial cells (black arrows), *RYK* CISH, 400×.

There was a rare occurrence (0/+) of *RYK* gene-signal-containing cells in the control group epithelium and connective tissue as the median value, while in the endothelium, the median was between no gene-signal-containing cells and a rare occurrence (0–0/+) of gene signals (Figure 6A). In the patient group, the median number of *RYK* gene-signal-containing cells was moderate (++) in the surface epithelium and endothelium, while in connective tissue, it was few to moderate (+/++) (Figure 6B). Statistically significant differences in the number of *RYK* gene-signal-containing cells were identified between controls and the patient group in the surface epithelium (U = 10.5, *p* = 0.005), in connective tissue (U = 12.0, *p* = 0.008), and in the endothelium (U = 13.0, *p* = 0.011).

**Figure 7:**
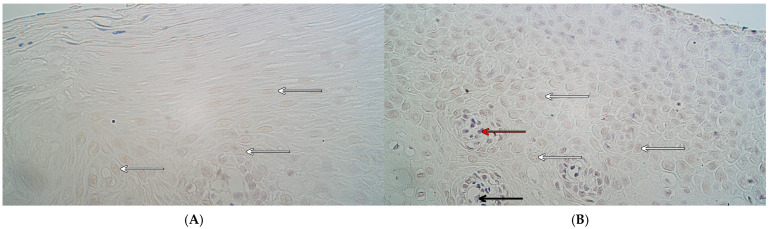
*PTX3* gene visualization in control tissue and cleft lip-affected tissue using chromogenic in situ hybridization. (**A**) Control tissue with a few *PTX3* gene-signal-containing epithelial cells (white arrows), *PTX3* CISH, 400×. (**B**) Cleft lip tissue with a few *PTX3* gene-signal-containing epithelial cells (white arrows) and a rare occurrence of *PTX3*-gene-signal-containing connective tissue cells (red arrow) and endothelial cells (black arrow), *PTX3* CISH, 400×.

*PTX3* gene signals had the following median values in the control group—between a rare occurrence and a few (0/+–+) in the epithelium, and a rare occurrence (0/+) in the connective tissue and endothelium (Figure 7A). In the patient group, a rare occurrence (0/+) of *PTX3* gene-signal-containing cells was identified in the epithelium and connective tissue as the median, while the median in the endothelium was 0 (Figure 7B). Statistically significant differences in the number of *PTX3* gene-signal-containing cells were not identified between controls and the patient group in any localization—not in the surface epithelium (U = 44.0, *p* = 0.970), not in connective tissue (U = 29.0, *p* = 0.235), and not in the endothelium (U = 31.0, *p* = 0.302).

The median values of gene-signal-containing cells in each group are available in Table 2.

### 2.3. Correlations

#### 2.3.1. Correlations in Control Group

The control group had 28 statistically significant correlations—17 very strong positive and 11 very strong negative correlations.

The strongest significant positive correlations in the control group were calculated between the number of RYK gene-signal-containing cells in the epithelium and MSX2 gene signals in the endothelium (r_s_ = 1.000; *p* < 0.001), between the number NFκB p65-positive endothelial cells and NFκB p65-positive connective tissue cells (r_s_ = 0.984; *p* < 0.001), between MSX2 gene-signal-containing endothelial cells and MSX2 gene-signal-containing connective tissue cells (r_s_ = 0.950; *p* = 0.004), between RYK gene-signal-containing epithelial cells and MSX2 gene-signal-containing connective tissue cells (r_s_ = 0.950; *p* = 0.004), and between CCL4-positive endothelial cells and NFκB p65-positive connective tissue cells (r_s_ = 0.922; *p* = 0.013). Most statistically significant positive correlations involved MSX2 and RYK gene-signal-positive cells—the number MSX2 gene-signal-containing cells was identified in seven statistically significant very strong correlations, while the number of RYK gene-signal-containing cells was in five correlations. In four cases, MSX2 and RYK gene signals correlated with each other. Regarding immunohistochemistry, NFκB p65 involvement was identified in five positive correlations; in four cases, NFκB p65 positively correlated with CCL4. Involvement of PTX3 gene-signal-containing cells was identified in two significant positive correlations. There were statistically significant positive correlations between MSX1-positive cells in the epithelium and MSX1-positive connective tissue cells (r_s_ = 0.874; *p* = 0.023), as well as a significant correlation between CCL4 and RYK-positive connective tissue cells (r_s_ = 0.822; *p* = 0.045).

Strongest statistically significant negative correlations in the control group were calculated between RYK gene-signal-containing connective tissue cells and NFκB p65-positive endothelial cells (r_s_ = −1.000; *p* < 0.001), between RYK gene-signal-containing connective tissue cells and NFκB p65-positive connective tissue cells (r_s_ = −0.984; *p* < 0.001), between RYK gene-signal-containing connective tissue cells and CCL4-positive endothelial cells (r_s_ = −0.904; *p* = 0.013), between MSX2 gene-signal-containing epithelial cells and RYK-positive connective tissue cells (r_s_ = −0.890; *p* = 0.018), and between MSX2 gene-signal-containing connective tissue cells and RYK-positive connective tissue cells (r_s_ = −0.867; *p* = 0.025). Statistically significant negative correlations mainly involved RYK gene-signal-positive cells, which were found in six significant negative correlations, MSX2 gene-signal-positive cells in five correlations, NFκB p65-positive cells in four correlations, CCL4-positive cells in four correlations, and RYK-positive cells in three correlations.

Correlations in the control group are summarized in Table 3.

**Figure 8:**
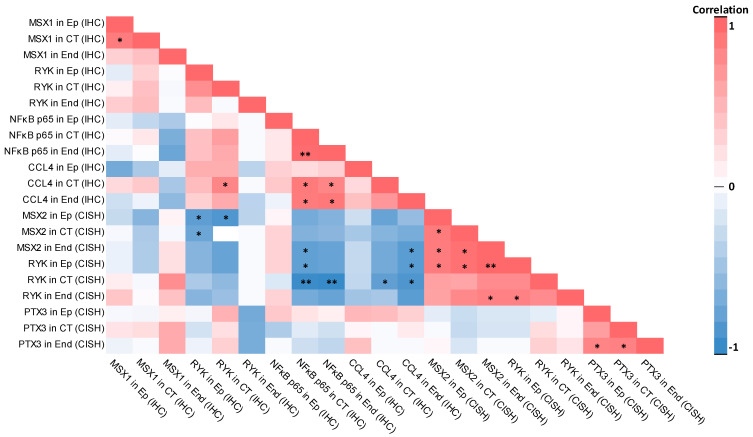
Correlation heat map between the evaluated factors in the control group. Note: statistically significant correlations are labeled with asterisks (*—*p* < 0.05; **—*p* < 0.001). Abbreviations: Ep—epithelium; CT—connective tissue; End—endothelium; IHC—immunohistochemistry; CISH—chromogenic in situ hybridization; MSX1—muscle segment homeobox 1; RYK—receptor-like tyrosine kinase; NFκB p65—nuclear factor kappa-light-chain-enhancer of activated B cells protein 65; CCL4—C-C motif chemokine ligand 4; MSX2—muscle segment homeobox 2; PTX3—pentraxin 3.

A correlation heat map of the evaluated factors in the control group tissue can be seen in Figure 8.

#### 2.3.2. Correlations in Patient Group

The patient group had 23 statistically significant correlations—22 were positive correlations (4 very strong, 9 strong, 9 moderate correlations) while only 1 correlation was negative.

The strongest positive correlations were identified between *RYK* gene-signal-containing cells in the endothelium and *RYK* gene-signal-containing cells in the connective tissue (r_s_ = 0.901; *p* < 0.001), between *RYK* gene-signal-containing connective tissue cells and RYK gene-signal-containing epithelial cells (r_s_ = 0.852; *p* < 0.001), between RYK gene-signal-containing endothelial cells and *RYK* gene-signal-containing epithelial cells (r_s_ = 0.822; *p* < 0.001), between NFκB p65-positive epithelial cells and RYK-positive epithelial cells (r_s_ = 0.802; *p* < 0.001), and between *PTX3* gene-signal-containing endothelial cells and *PTX3* gene-signal-containing epithelial cells (r_s_ = 0.789; *p* < 0.001). Statistically significant positive correlations involved *PTX3* gene-signal-containing cells, which were seen in seven correlations, NFκB p65-positive cells in seven correlations, MSX1-positive cells in six correlations, RYK-positive cells in five correlations, CCL4-positive cells in four correlations, *RYK* gene-signal-containing cells in three correlations, and *MSX2* gene-signal-containing cells in two correlations.

The only statistically significant moderate negative correlation in the patient group was identified between *MSX2* gene-signal-containing connective tissue cells and CCL4-positive endothelial cells (r_s_ = −0.585; *p* = 0.019).

Correlations in the patient group are summarized in Table 4.

**Figure 9:**
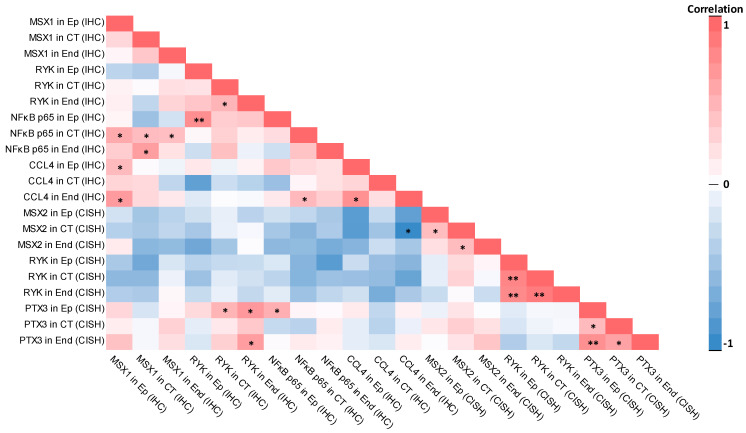
Correlation heat map between the evaluated factors in the patient group. Note: statistically significant correlations are labeled with asterisks (*—*p* < 0.05; **—*p* < 0.001). Abbreviations: Ep—epithelium; CT—connective tissue; End—endothelium; IHC—immunohistochemistry; CISH—chromogenic in situ hybridization; MSX1—muscle segment homeobox-1; RYK—receptor-like tyrosine kinase; NFκB p65—nuclear factor kappa-light-chain-enhancer of activated B cells protein 65; CCL4—C-C motif chemokine ligand 4; MSX2—muscle segment homeobox 2; PTX3—pentraxin 3.

A correlation heatmap for the evaluated factors in the patient group is visualized in Figure 9.

## 3. Discussion

### 3.1. Immunoreactivity of MSX1, NFκB p65, and CCL4 Proteins Was Significantly Decreased in Cleft Lip Connective Tissue and Endothelium but Not in Surface Epithelium

Our study revealed that MSX1, NFκB p65, and CCL4-positive cells were significantly decreased in cleft lip-affected connective tissue and endothelium, although the immunoreactivity in the surface epithelium did not differ from controls. This indicates that the decrease in MSX1, NFκB p65, and CCL4 proteins might be involved in postnatal cleft lip morphopathogenesis, while the exact mechanisms are relatively unclear.

A decrease in MSX1 as seen in cleft lip connective tissue and endothelium corresponds with existing information in other studies. Knockout mutations of the *MSX1* gene, which encodes the MSX1 protein, have been associated with cleft lip and palate formation in animal models [46], and human studies have also suggested that *MSX1* gene mutations might be associated with a cleft lip and palate [47,48]. The *MSX1* gene also regulates tissue proliferation during lip development, and a significant decrease in the MSX1 protein causes the cleft lip phenotype in mice [49]. A decrease in MSX1 in cleft lip tissue most likely is an important aspect of human cleft lip morphopathogenesis even postnatally, as observed from our study results. Interestingly, MSX1 deficiency disrupts the growth of the medial nasal process in mice, and this is exacerbated by hypoxia during pregnancy [50], which might mean that a similar process could also be happening during human cleft lip morphopathogenesis in developing craniofacial connective tissue and blood vessels, although additional research is needed to confirm this suggestion. MSX1 expression in endothelial cells has been observed in previous studies [51,52], and the decrease in MSX1-positive endothelial cells of cleft lip-affected tissue might suggest that MSX1 deficiency disturbs normal blood vessel growth in cleft-affected tissue while interacting with other factors. Although MSX1-positive cells were identified in cleft-affected surface epithelium, the immunoreactivity did not differ from the controls, which suggests that the connective tissue and vasculature of the cleft lip are more important areas of MSX1 interactions in cleft lip morphopathogenesis.

The NFκB p65 and CCL4 proteins are involved in regulation of inflammation and immune function. Although the presence of orofacial clefts is associated with increased pro-inflammatory cytokines and the presence of inflammation in oral mucosa [53], our study results suggest that NFκB p65 and CCL4-positive cells in cleft lip tissue were decreased in comparison to control tissue, which was unexpected. The exact mechanism for this result is unclear, but it could be associated with the specific functions of NFκB p65 and CCL4 in tissue as well as the characteristics of the research group. The cleft lip study group contained patients who did not have significant inflammation present in their tissue (no signs of leukocyte infiltration, no lymphoid follicles or fibrotic changes in connective tissue). As the patient group was relatively small and the presence of inflammatory changes in tissue was limited, this could explain why the relative number of NFκB p65 and CCL4-positive cells was decreased in comparison to controls. NFκB p65 stimulates neutrophil adhesion and transmigration in tissue [54], while CCL4 enhances the adhesion of T lymphocytes to endothelial cells [55,56] and chemoattraction of macrophages and other leukocytes [57]. The fact that inflammation and the presence of immune cells was limited or not present in most patient tissue samples could explain the significant differences between the research groups in both NFκB p65 and CCL4 immunoreactivity.

Further details that might cause the decreased presence of NFκB p65 and CCL4-positive cells are other factors and signaling pathways that might be disturbed in cleft-affected tissue. For example, RYK, which functions in the WNT signaling pathway, can inhibit NFκB signaling, which limits inflammation, as seen in lung mesenchyme [19]. This interaction might be similar in cleft lip tissue as well. The exact interaction within cleft lip tissue might be affected by other factors and might not be fully explained by the activity of the *RYK* gene and protein alone, as our research results indicate an increased presence of RYK gene-signal-containing cells and a decrease in RYK protein-containing cells in cleft lip tissue.

The lack of statistically significant difference in NFκB p65 and CCL4-positive epithelial cells between controls and cleft lip patients might indicate that the surface epithelium most likely is not directly connected with postnatal changes that affect cleft lip tissue homeostasis.

### 3.2. RYK Protein Immunoreactivity Was Significantly Decreased Only in Cleft Lip-Affected Connective Tissue

The number of RYK protein-positive connective tissue cells was significantly decreased in comparison to controls. RYK functions as a part of the WNT signaling pathway and can interact with different WNT ligands [17]. Downregulation of RYK has been associated with decreased cell proliferation and cell renewal, as seen in mesenchymal stromal cells of the red bone marrow [58]. It could be proposed that a similar mechanism of RYK dysfunction might be present in the ectomesenchymal cells of the craniofacial region as well, which would later form the connective tissue of the developing lip. Disrupted WNT signaling as well as dysfunction of RYK have been associated with orofacial cleft formation in previous studies [59]. Why the significant decrease in RYK protein-positive cells was observed only in cleft lip connective tissue and not in the surface epithelium or endothelial cells remains uncertain, as RYK immunoreactivity was observed in all patient and control tissue samples in each of the localizations. This might mean that cleft lip connective tissue postnatally is the most promising localization for further research to analyze disturbed WNT signaling, WNT ligands, RYK, and other WNT receptors in more detail, to clarify the exact morphopathogenetic mechanisms. RYK is also indirectly connected with other signaling pathways like NFκB signaling [19], which complicates the exact role and assessment of this protein in cleft tissue homeostasis.

### 3.3. The Number of MSX2 and RYK Gene-Signal-Containing Cells Was Significantly Increased in Cleft Lip Tissue While the Number PTX3 Gene-Signal-Containing Cells Did Not Differ from Controls

The number of *MSX2* and *RYK* gene-signal-containing cells was significantly increased in the cleft lip in all three evaluated tissue types (epithelium, connective tissue, and endothelium) in comparison to the control group.

Interestingly, the *MSX2* gene together with the *MSX1* gene are downstream targets of the WNT/β-catenin signaling pathway during lip development as seen in mice models, and downregulation of both *MSX1* and *MSX2* genes prevents the correct growth and fusion of orofacial primordia, causing the cleft lip [60]. Our study results indicate that in humans with a cleft lip, the *MSX2* gene is significantly more active postnatally in cleft lip tissue than relatively healthy oral mucosa tissue, which might be a compensatory mechanism, possibly caused by a disrupted WNT signaling pathway within cleft-affected tissue, which is indicated by the changes in other evaluated factors in cleft tissue, like the *RYK* gene and RYK protein. The fact that the presence of the MSX1 protein, which was also analyzed in our study, was decreased in cleft lip tissue while the *MSX2* (which has similar functional activity to *MSX1*) gene-signal-containing cells still significantly more present in cleft lip tissue might mean that the activity of genes does not always correlate with later translation of specific gene-coded proteins. Expression of the *MSX2* gene postnatally has been seen in other orofacial tissue localizations like the dental pulp [61], but information about *MSX2* gene activity in lip tissue postnatally remains limited. Intriguingly, the presence the MSX2 protein assessed with IHC in a previous study with human cleft lip tissue samples did not show statistically significant differences between controls and patients, while MSX2 protein-containing cells were not detected in the surface epithelium and were barely detectable in connective tissue of both patients and controls [62]. This discrepancy between the significant presence of potentially active *MSX2* genes in cleft lip tissue and barely any MSX2 protein-positive cells in the same type of cleft-affected tissue indicates that additional regulatory or compensatory mechanisms are present, which might affect gene transcription and later protein translation within cleft lip tissue cells, and that these mechanisms are not present in relatively healthy oral cavity tissue. Further research assessing MSX2 gene transcription regulation and protein translation in cleft tissue could clarify the exact role of the MSX2 gene in cleft mrophopathogenesis.

Another finding was that the number *RYK* gene-signal-containing cells was increased in cleft lip tissue in comparison to controls. As discussed previously, the RYK protein is a part of the non-canonical WNT signaling pathway [63,64]. The *RYK* gene is expressed in certain tissue like nervous tissue [65] and hematopoietic stem cells [66] while information about *RYK* gene expression in oral cavity tissue is limited, although *RYK* gene mutations have been associated with a cleft lip and palate previously, which indicates its involvement in orofacial tissue growth and differentiation [22]. Our study findings indicate that the *RYK* gene might be more functionally active in cleft lip tissue, possibly due to disturbed signaling pathways and tissue homeostasis characteristics, similarly to what is seen in the case of the *MSX2* gene.

Intriguingly, while the number of *RYK* gene-signal-containing cells was increased, the number of RYK protein-containing cells in cleft lip connective tissue and endothelium was decreased in comparison to controls. This discrepancy might be explained by the fact that gene transcription and subsequent translation are processes that can be regulated by transcription factors, translation factors, and other regulatory mechanisms [67]. An important aspect is individual gene expression variation seen in the human population due genetic variations and translation regulation, which affects protein levels in cells [68]. This might mean that disturbances and complicated interactions in different signaling pathways like WNT signaling and others in cleft lip tissue might directly or indirectly affect both the transcription of the *RYK* gene as well as translation of the RYK protein within cells, which could explain the findings seen in our study. The exact mechanism of these processes might not be exactly clear, but this could be a unique direction for future research into cleft morphopathogenesis.

The number of *PTX3* gene-signal-containing cells did not differ between cleft lip tissue and controls. *PTX3* gene expression is typically seen in inflammatory conditions [69,70], and as orofacial clefts have been associated with local tissue inflammation [53], the increased number of *PTX3* gene-signal-containing cells should have been expected in the cleft lip patient group. The possible reason why no significant difference was seen between the groups could be the relative lack of excessive inflammation signs present in cleft lip tissue samples used in this study. Without active inflammation and the presence of a significant number of immune cells in cleft-affected tissue, the relative number of *PTX3* gene-signal-containing cells most likely would be similar to the number seen in control tissue. This most likely also affected the presence of NFκB p65 and CCL4 proteins in cleft lip tissue, as discussed previously.

### 3.4. Statistically Significant Correlations Between Factors Were Mostly Different Within Control and Patient Groups

We identified 28 statistically significant correlations (17 positive, 11 negative) between protein-positive/gene-signal-positive cells in the control group, while 23 statistically significant correlations (22 positive, 1 negative) were identified in the cleft lip patient group. Six positive correlations between the same factors were identified in both groups—between CCL4 in endothelium and NFκB p65 in connective tissue, between *MSX2* in connective tissue and *MSX2* in epithelium, between *MSX2* in endothelium and *MSX2* in connective tissue, between *RYK* in endothelium and *RYK* in epithelium, between *PTX3* in endothelium and *PTX3* in epithelium, and between *PTX3* in endothelium and *PTX3* in connective tissue (all CISH). All other statistically significant correlations did not overlap between the control group and the patient group, which indicates significant differences in tissue homeostasis, most likely due to disturbed molecular signaling pathways within cleft-affected tissue.

The correlation profile between controls and patients had some similarities as well as multiple differences, which especially stood out in the relatively large number of statistically significant negative correlations within the control group that were not present in the patient group.

Statistically significant negative correlations in the control group were mostly identified between *MSX2* gene-signal-containing cells and RYK protein-containing cells between the *MSX2* gene and NFκB p65 protein/CCL4 protein and between *RYK* gene-signal-containing cells and NFκB p65 and CCL4 protein-containing cells. The exact mechanism might relate to two signaling pathways—WNT signaling and NFκB signaling. As discussed previously, the *MSX2* gene is a downstream target of the activated WNT/β catenin signaling pathway [71], and the RYK protein functions in the non-canonical WNT signaling pathway [72]. The RYK protein and *MSX2* gene do not interact with each other directly but certain WNT signaling pathway molecular cascade mechanisms most likely try to maintain proper tissue homeostasis and growth regulation within relatively healthy lip tissue by eventually affecting both the RYK protein as well as the *MSX2* gene. On the other hand, the NFκB signaling pathway regulates local tissue inflammation and is intertwined with WNT/β-catenin signaling, where both positive and negative regulation are possible on each other [73]. Activated NFκB signaling also stimulates CCL4 expression through tumor necrosis factor α (TNF-α) [74], so inhibited NFκB signaling activity might also suppress CCL4 production. The negative correlations between these factors in the control group indicate that these signaling pathways strictly regulate each other for proper tissue function. The lack of these statistically significant negative correlations in postnatal cleft lip tissue indicates significant disturbances in signaling pathway regulation due to the presence of the cleft.

The only statistically negative correlation seen in the cleft lip patient group was identified between the MSX2 gene in connective tissue (CISH) and the CCL4 protein in the endothelium (IHC), which was not present in the control group. This negative correlation might again have developed due to disturbances in WNT and NFκB signaling pathways, which are interconnected, as discussed previously [73]. The exact interaction most likely is indirect and mediated by multiple other factors within disturbed cleft lip tissue signaling pathways.

Positive correlations in the control group mainly involved *MSX2* and *RYK* gene-signal-containing cells, which correlated with each other in four correlations. This again emphasizes the role of a disturbed WNT signaling pathway where both MSX2 and RYK are indirectly connected [71,72], although the correlation between gene-signal-positive cells is now positive and not negative as it was with the RYK protein. This most likely means that the *RYK* gene activity and the formation of RYK protein are dependent on separate factors, most likely still connected to the WNT signaling pathway. Similar positive correlations in the control group were observed, with positive correlations between the NFκB p65 protein and CCL4 protein, again possibly due to the interaction in the NFκB signaling pathway [74]. In multiple cases, the same factor correlated with itself but in a different tissue location; for example, *PTX3* gene-signal-containing cells in the endothelium correlated with *PTX3* in the epithelium and connective tissue. Similarly, a correlation between the MSX1 protein in the epithelium and MSX1 protein in the connective tissue was also found, which emphasizes the interaction and signaling activity between different tissue types of the lip.

In cleft lip patients, most statistically significant positive correlations involved the following factors: the *PTX3* gene and NFκB p65 protein, then MSX1 protein-positive cells, RYK protein-positive cells, the CCL4 protein, the *RYK* gene, and then *MSX2* gene-signal-containing cells. This arrangement is quite different from correlations seen in the control group. First, *PTX3* gene-signal-containing cells correlated with the RYK protein and NFκB p65-positive cells, which was not seen in the control group. Interaction between PTX3 and WNT signaling has previously been described [75] but information about the connection between PTX3 and non-canonical WNT signaling is not fully clarified in previous research. This indicates that, although the presence of inflammation in the cleft lip patient group was not present, which would affect and increase the number of *PTX3* gene-containing cells to significant levels, this factor still significantly correlated with the RYK and NFκB p65 proteins. This might mean potential direct or indirect interaction between these factors and possible regulation of local inflammation and immune function. Another group of positive correlations that stood out in the cleft lip patient group was between NFκB p65 and MSX1, which were not present in the control group. The *MSX1* gene in mice has multiple enhancer elements with three potential NFκB-binding sites [76], which could mean that a similar interaction could be present in humans as well. Disturbed NFκB signaling in cleft lip tissue could explain the correlation between NFκB p65 and MSX1 proteins. There were correlations where the same factor correlated with itself in different tissue localizations; for example, this was seen for the *PTX3* and *RYK* genes in the epithelium, connective tissue, and endothelium, indicating a strong interaction between each tissue and cell type in gene activity.

The evaluated correlations in both patients and controls indicate the presence of complicated regulatory mechanisms that maintain and regulate local tissue homeostasis. The cleft lip tissue is characterized by disrupted molecular regulatory mechanisms, as seen in different and unique correlations between evaluated factors that were not found in controls.

### 3.5. Limitations of This Study

A limitation of this study was the use of only two methods (IHC and CISH) to determine the relative number of protein-containing and gene-signal-containing cells, respectively, within the patient and control groups. Additional methods could provide additional information to improve the analysis and significance of each evaluated factor and their possible role in cleft lip morphopathogenesis; for example, radioimmunohistochemistry and enzyme-linked immunosorbent assay (ELISA) could help to determine exact protein concentrations within tissue, and other methods, such as transcriptome analysis and genetic analysis, could provide more detailed analyses about individual characteristics and molecular variations in each patient. The semiquantitative counting method cannot be used to interpret the exact concentrations of proteins within cells, and it is also just one of the possible counting methods that can be used to interpret the presence of protein-containing/gene-signal-containing cells in cleft lip and control tissue.

An important limitation is the relatively small size of the patient and control groups, which can affect data analysis and complicate the generalization of results. In relatively small patient and control groups, patients’ individual characteristics, data outliers, and other confounding factors can significantly affect result interpretation, which means that results and conclusions must be assessed with caution. Access to human tissue in cleft research is very limited due to ethical considerations, as this tissue is obtained from children with the consent of their parents/legal guardians. Additionally, another difficulty is obtaining enough relatively healthy oral mucosa tissue to form a control group due to the same ethical aspects. The age differences between control and patient group individuals are also a significant factor that can affect the assessment of protein-positive and gene-signal-positive cells in oral cavity tissue, as the age of individuals can influence the expression of certain proteins as well as gene activity in tissue. Although both patient and control tissues were gathered before or during the age of primary dentition, the variations in patient age as seen in the control group as well as the patient group can be a considerable factor that might affect the exact presence and activity of certain proteins/genes in children’s oral cavity tissue.

Nonetheless, this research is unique as it was performed with human tissue, while most orofacial cleft morphological research is typically carried out using animal models and the results are not always directly transferable to humans. Datasets from animal models could be valuable in analyzing different factors in cleft lip morphopathogenesis but not all data can be used in humans due to differences in species, genetic, and molecular characteristics.

### 3.6. Clinical Application of Data and Possible Future Research Directions

The information gathered in this study has provided additional insight about the presence and distribution of MSX1, RYK, NFκB p65, and CCL4 proteins and *MSX2*, *RYK*, and *PTX3* genes in human cleft lip tissue. This study has identified certain possible interactions between the evaluated genes/proteins, indicating that certain immunomodulatory factors and certain cleft candidate genes or their coded proteins might continue their functional activity in cleft lip tissue postnatally, while others like the *PTX3* gene, which is typically associated with local tissue inflammation, most likely are not a significant factor in cleft lip morphopathogenesis. In the clinical context, the knowledge about inflammatory/immunomodulatory factors in cleft-affected tissue might help in understanding the differences in local inflammation before a surgical intervention and could form the basis for additional pharmacological methods that specifically regulate the local activity of the evaluated factors, which could help in wound healing in complicated surgical cases. For example, if excessive activity of certain signaling pathways like NFκB signaling in cleft tissue is noted, then certain medications could help to normalize tissue homeostasis before cleft-correcting surgery and could help to decrease the risk of complications after the intervention. This, of course, can only be speculated as the knowledge about cleft lip morphopathogenesis postnatally is still not full.

Understanding cleft candidate genes and their coded proteins postnatally in cleft-affected tissue could be useful for clarifying cleft pathogenesis, which is still not fully understood in most non-syndromic orofacial cleft cases. It is not possible to develop targeted and safe cleft pharmacological treatment and prophylaxis if the exact cleft formation pathogenetic mechanisms are not fully known. This research work hopefully is a small step in untangling the complicated network of mechanisms that form clefts and affect cleft tissue growth postnatally. Determining the exact interactions between the evaluated factors and their exact role in cleft tissue is beyond the scope of this research work and additional studies are necessary. Future research could focus on clarifying the role of specific signaling pathways in orofacial cleft morphopathogenesis, like WNT signaling, NFκB signaling, and others, which might be the most promising directions for the development of cleft pharmacological treatment or even cleft prophylaxis in humans.

## 4. Materials and Methods

### 4.1. Patient Group and Control Group Characteristics

Tissue material from both controls and patients was obtained for research in the Cleft Lip and Palate Centre of the Institute of Stomatology of Riga Stradins University (RSU) as a voluntary donation with written agreement from parents of patients/controls. Only the minimum amount of soft upper lip tissue (approximately 1 mm^3^) was obtained during lip plastic surgery for research purposes. The Millard method was used during primary lip plastic surgery for unilateral cleft lip patients [77], while for bilateral cleft lip patients, the modified Veau method was used [78]. Tissue morphological analysis was conducted in the RSU Institute of Anatomy and Anthropology. The RSU Research Ethics Committee provided the following permissions—1st permission dated 22 May 2003 (code 22.05.2003) and 2nd permission (Nr. 6-1/10/11) dated 24 September 2020. This study was performed in accordance with the newest revision of the Declaration of Helsinki.

Cleft lip tissue was gathered from individuals based on the following inclusion criteria:

- Diagnosis of cleft lip;
- Cleft lip surgery performed before/during the age of primary dentition;
- No malignancy, excessive inflammation, fibrosis, or any other pathological change in the soft tissue of the lip present.

In total, tissue samples from 15 individuals with cleft lips were investigated. The average age of cleft lip patients was 4.3 ± 1.1 months, ranging from 3.5 months up to 7.0 months. Eight individuals were boys and seven girls. Twelve individuals had a unilateral cleft lip (ten patients had it on the left side, two patients on the right side), and three individuals had a bilateral cleft lip. Fourteen patients did not have a cleft lip or palate in the family anamnesis, but one patient had a positive family history for orofacial clefts. All patient tissue samples were taken before/during the age of primary dentition.

Inclusion criteria for individuals in the control group were the following:

- Individuals without cleft lip and palate;
- No cleft lip and palate in family anamnesis;
- Individuals before/during the age of primary dentition (or as close as possible);
- No malignancy, excessive inflammation, fibrosis, or any other pathological change in the soft tissue of the lip present.

Control group consisted of 6 individuals. Tissue material from 5 individuals was gathered from the historical collection of the RSU Institute of Anatomy and Anthropology—4 of them were newborns (2 died from umbilical cord strangulation while the other 2 died from sudden infant death syndrome; the 5th individual died during medical abortion during the 24th gestation week due to a maternal health condition). One control tissue sample was obtained from a child with hypertrophic upper lip frenulum from a 3-year-old patient. Five controls were girls while one individual was a boy. None of the controls had a cleft lip or palate, and this pathology was not found in the family anamnesis. All control tissue samples were taken before/during the age of primary dentition.

All tissue samples were fixed in Stefanini solution after retrieval. Tissue was further prepared in the Laboratory of Morphology of the RSU Institute of Anatomy and Anthropology. Tyrode’s solution was used to wash tissue samples for 24 h. Tissue was dehydrated in alcohol solution. Next, degreasing with xylene for 30 min was performed. Afterward, tissue was embedded in paraffin. Paraffin blocks were cut with a semi-automatic rotary microtome (Leica RM2245, Leica Biosystems Richmond Inc., Richmond, IL, USA). Tissue was later prepared for immunohistochemistry (IHC) to identify protein-positive cells, and chromogenic in situ hybridization (CISH) to identify the presence of gene-signal-containing cells.

### 4.2. Immunohistochemistry (IHC) and Chromogenic In Situ Hybridization (CISH)

IHC was used to determine the number of MSX1, RYK, NFκB p65, and CCL4-positive cells in both study groups. The standard streptavidin and biotin immunostaining method [79] was employed. IHC based on technical specifications set by manufacturers was performed with the following antibodies:

- MSX1 antibodies (LS-C47382/11448, rabbit, polyclonal, dilution 1:200, LifeSpan BioSciences Inc., Seattle, WA, USA);
- RYK antibodies (orb38371, rabbit, polyclonal, dilution 1:100, Biorbyt Ltd., Cambridge, UK);
- NFκB p65 antibodies (orb37069, rabbit, polyclonal, dilution 1:100, Biorbyt Ltd., Cambridge, UK);
- CCL4 antibodies (ab235978, rabbit, polyclonal, dilution 1:100, Abcam Inc., Cambridge, UK).

CISH was performed to identify and visualize the presence of MSX2, RYK, and PTX3 gene signals in tissue samples by using specific peptide nucleic acid probes [80]. The ZytoDot2C CISH Implementation Kit (ZytoVision GmbH, Bremerhaven, Germany) was used with the following probes:

- *MSX2* probe (MSX2-20-DIG, Empire Genomics Corp., Williamsville, NY, USA);
- *RYK* probe (RYK-20-DIG, Empire Genomics Corp., Williamsville, NY, USA);
- *PTX3* probe (PTX3-20-DIG, Empire Genomics Corp., Williamsville, NY, USA).

The following procedure for CISH implementation was used [7]. Slides were pretreated with standard laboratory methods before denaturation and hybridization were performed. A 10 μL probe was added to the slides, and clean coverslips were used on top of them while trying to prevent the formation of air bubbles. Afterwards, slides were kept at 79 °C for 5 min, later transported to a humidity chamber, and then hybridized at 37 °C for 24 h. Later, coverslips were taken off from slides, and each slide was put in Wash Buffer SSC at 80 °C for 5 min. Afterwards, slides were rinsed with distilled water and put in Wash Buffer TBS. Next, 1–2 drops of Anti-Digoxigenin/Dinitrophenol (Anti-DIG/DNP) Mix were put on every slide. Incubation in the humidity chamber at 37 °C for 15 min was performed. Later, slides were again put in freshly made Wash Buffer TBS three times with the duration of 1 min. AP-Red solution was produced by mixing 1ml Alkaline Phosphatase Red (AP-Red) Solution B and 1 drop (30 μL) of AP-Red Solution A, and 1–2 drops of this mixture were put on to every slide and kept for 10 min at room temperature. While waiting, Horseradish Peroxidase Green (HRP-Green) Solution was then produced by mixing 1 mL of HRP-Green Solution B and 2 drops (2 × 20 μL) of HRP-Green Solution A. After the 10 min of waiting, slides were rinsed with distilled water for 2 min, and 1–2 drops of HRP-Green Solution were put on slides. Afterwards, slides were kept again at room temperature for 10 min. Again, rinsing with distilled water for 2 min was performed, and slides were colored with Nuclear Blue Solution for 2 min. Afterwards, each specimen was put into a staining jar, rinsed with cold running water for 2 min, dehydrated with 100% ethanol, and incubated in xylene afterwards. Coverslips were put back again on slides, and probe signals could be visualized under brightfield light microscopy. Two brown-colored dots were expected in normal cell nuclei if cells in tissue were in the interphase or metaphase of the cell cycle.

Slides prepared by IHC and CISH were examined with brightfield light microscopy. Microphotographs of slides were captured with a Leica DC 300F digital camera (Leica Microsystems Digital Imaging, Cambridge, UK). Afterwards, image processing was conducted with the Image Pro Plus program (Media Cybernetics, Inc., Rockville, MD, USA).

### 4.3. Semiquantitative Counting Method and Data Analysis

The semiquantitative counting method was used to determine the relative frequency of protein-containing cells (for slides prepared with IHC) and gene-signal-containing cells (for slides prepared with CISH) [81]. Slides were analyzed by two independent researchers in at least five separate visual fields in each slide to determine the semiquantitative score for the specific slide. The median value of the semiquantitative score for each slide assessed by both researchers was used for data analysis. Cells were analyzed in the following tissue localizations, which were identifiable in each slide for both controls and patients—in the surface epithelium (Ep), connective tissue (CT), and endothelium (End).

The following semiquantitative cell count values were implemented in this study:

- 0—no protein-containing/gene-signal-containing cells in the visual field (0.0%);
- 0/+—a rare occurrence of protein-containing/gene-signal-containing cells in the visual field (0.0–12.5%);
- +—a few protein-containing/gene-signal-containing cells in the visual field (12.5–25.0%);
- +/++—few to moderate protein-containing/gene-signal-containing cells in the visual field (25.0–37.5%);
- ++—a moderate number of protein-containing/gene-signal-containing cells in the visual field (37.5–50.0%);
- ++/+++—moderate to numerous protein-containing/gene-signal-containing cells in the visual field (50.0–62.5%),
- +++—numerous protein-containing/gene-signal-containing cells in the visual field (62.5–75.0%),
- +++/++++—numerous to abundant protein-containing/gene-signal-containing cells in the visual field (75.0–87.5%);
- ++++—abundant protein-containing/gene-signal-containing cells in the visual field (87.5–100.0%).

Data statistical analysis was performed with both descriptive and analytical methods. The median values of the semiquantitative count for protein-containing cells/gene-signal-containing cells were determined. The Mann–Whitney U test was used to assess if there were statistically significant differences between controls and patients in the number of protein-containing/gene-signal-containing cells. Spearman’s correlation coefficient (r_s_) was used to calculate correlations between the evaluated proteins/genes with the following values: 0.0–0.2—very weak correlation, 0.2–0.4—weak correlation, 0.4–0.6—moderate correlation, 0.6–0.8—strong correlation, 0.8–1.0—very strong correlation. Statistical analysis was performed with Statistical Product and Service Solutions (SPSS) Statistics version 29.0.0.0 (IBM Company, Chicago, IL, USA). Statistical significance of every calculation was determined with a *p*-value of < 0.05.

## 5. Conclusions

1. Immunoreactivity of MSX1, NFκB p65, and CCL4 proteins was significantly decreased in cleft lip connective tissue and endothelium but not in surface epithelium; a decrease in MSX1 has been previously associated with disturbed growth and fusion of the developing upper lip primordia, while the decrease in NFκB p65 and CCL4 proteins could be explained by the characteristics of the patient group and lack of active inflammation within tissue.
2. RYK protein immunoreactivity was significantly decreased only in cleft lip-affected connective tissue, possibly due to a disrupted WNT signaling pathway in cleft lip tissue.
3. The number of *MSX2* and *RYK* gene-signal-containing cells was significantly increased in cleft lip tissue, probably due to WNT signaling disturbances and gene activation in cleft-affected tissue.
4. The number of *PTX3* gene-signal-containing cells did not differ from controls, possibly due to characteristics of the patient group and the lack of active inflammation in tissue.
5. Statistically significant correlations between factors were mostly different within the control and patient groups, with some minor overlap being present; negative correlations predominantly were identified in the control group and not the cleft lip tissue group, indicating a disturbance of factor regulation within cleft lip tissue; positive correlations in the cleft lip group mainly involved the *PTX3* gene, NFκB p65, and MSX1 protein, which was not seen in the control group, again possibly due to disturbed signaling pathways in cleft lip tissue.

## Figures and Tables

**Table 1:** Semiquantitative evaluation of MSX1, RYK, NFκB p65, and CCL4 immunoreactive cells in controls and cleft lip patients.

Group	Number	MSX1 (IHC)			RYK (IHC)			NFκB p65 (IHC)			CCL4 (IHC)		
		Ep	CT	End	Ep	CT	End	Ep	CT	End	Ep	CT	End
**Controls**	1	++	++	++	+++	+++	++	++	++	++	++	++	++
	2	+++	++/+++	++	+++/++++	+++	+++/++++	++	++	++	+/++	++	+/++
	3	+++/++++	++/+++	+++	+++	+++	+++	++/+++	+/++	+/++	+/++	++	+
	4	+++	++	+/++	++/+++	++/+++	+++	++/+++	++	++	+	++	+/++
	5	++	++	+/++	+++/++++	+++/++++	+++	+++	+++	++/+++	++/+++	++/+++	++
	6	+/++	+/++	++	+++	++/+++	+++	++/+++	+	+/++	++	+	+
	**Median**	**++/+++**	**++**	**++**	**+++**	**+++**	**+++**	**++/+++**	**++**	**++**	**+/++–++**	**++**	**+/++**
**Patients**	1	++/+++	0/+	+	+++	++/+++	+++	++/+++	+	+	++	0/+	+
	2	++	++	+/++	++	+++	++/+++	+/++	+	+/++	+	+	0/+
	3	+/++	0/+	0/+	+++/++++	++/+++	+++	+++	0/+	0/+	+/++	0/+	0/+
	4	++	+	+	+++	++	++	++/+++	+	0	+	0/+	0/+
	5	++/+++	+/++	+	+++	++	+++	++	+	+	+/++	+	0/+
	6	++/+++	+	+	++/+++	++	+++	++/+++	+	0/+	++/+++	+/++	+
	7	++	+/++	+	+++	+/++	++	++	0/+	+	++/+++	+	+
	8	++	+	+	+++/++++	++/+++	++/+++	+++	+	+	++	0	0/+
	9	+/++	0/+	+	+++	++/+++	+++	++	0/+	0/+	+/++	+	0
	10	++/+++	+/++	+	+++/++++	+++	+++	+++	+/++	+	++/+++	+	+
	11	++/+++	+/++	0/+	++	++/+++	++	++	+	+/++	++	++	+
	12	+/++	+	0/+	++/+++	+	++	+/++	0/+	0/+	+	0/+	0
	13	++	+/++	+/++	+++	++	++/+++	++	+/++	+	++	+	+
	14	++	0/+	0/+	++	+	+	+/++	0/+	0	+/++	+	0/+
	15	++	+	0/+	+++	++	++	++/+++	+	+	++	++	0/+
	**Median**	**++**	**+**	**+**	**+++**	**++**	**++/+++**	**++**	**+**	**+**	**++**	**+**	**0/+**
**U**		30.5	5.0	2.0	33.5	12.0	23.5	35.5	7.0	2.0	44.0	10.5	6.0
***p***		0.267	0.001	<0.001	0.381	0.008	0.095	0.470	0.002	<0.001	0.970	0.005	0.001

Abbreviations: MSX1—muscle segment homeobox-1; RYK—receptor-like tyrosine kinase; NFκB p65—nuclear factor kappa-light-chain-enhancer of activated B cells protein 65; CCL4—C-C motif chemokine ligand 4; IHC—immunohistochemistry; Ep—epithelium; CT—connective tissue; End—endothelium; U—Mann–Whitney U test value; *p*—*p*-value; 0—no protein-positive cells in the visual field; 0/+—a rare occurrence of protein-positive cells in the visual field; +—a few protein-positive cells in the visual field; +/++—few to moderate protein-positive cells in the visual field; ++—a moderate number of protein-positive cells in the visual field; ++/+++—moderate to numerous protein-positive cells in the visual field; +++—numerous protein-positive cells in the visual field; +++/++++—numerous to abundant protein-positive cells in the visual field.

**Table 2:** Semiquantitative evaluation of *MSX2*, *RYK*, and *PTX3* gene-signal-containing cells in controls and cleft lip patients.

Group	Number	*MSX2* (CISH)			*RYK* (CISH)			*PTX3* (CISH)		
		Ep	CT	End	Ep	CT	End	Ep	CT	End
**Controls**	1	0	0	0	0/+	0	+	++	+/++	0
	2	0	0	0	0/+	0	0	0/+	0	0
	3	0/+	0/+	+	+	+	+	+/++	+/++	0/+
	4	+	0/+	+	0/+	0/+	0/+	0/+	0	+
	5	0	0	0	0	0	+	0/+	0/+	0
	6	+	+	+/++	+	0/+	0/+	0/+	0/+	+
	**Median**	**0/+**	**0–0/+**	**0–0/+**	**0/+**	**0/+**	**0–0/+**	**0/+–+**	**0/+**	**0/+**
**Patients**	1	+	+/++	++	++	++	+	+	+	+
	2	++	+	++	++/+++	++	+	+/++	+	++
	3	+/++	+	+	+	0/+	+/++	0/+	0/+	+/++
	4	+	+	++/+++	++	++	0/+	0	0	+
	5	++	++	0/+	0/+	0	+	+/++	+	++
	6	+/++	+/++	+++	++/+++	++	++	0/+	+	+/++
	7	0/+	0/+	++	+/++	++	0/+	+	0	0/+
	8	++	0/+	+++	++/+++	++/+++	+	0/+	0	++
	9	+/++	+	+++	++/+++	++	0/+	0/+	0	+/++
	10	+	+	++	+	+	+	0/+	0/+	+
	11	+	+	+/++	+	+	0/+	0	0	+
	12	+/++	+/++	++/+++	++/+++	++	0	0	0	+/++
	13	0	0	0	0	0	0	0	0	0
	14	++	++	++/+++	+/++	+	0	0/+	0	++
	15	+	0/+	+/++	+/++	+	0/+	0	0	+
	**Median**	**+/++**	**+/++**	**+**	**++**	**+/++**	**++**	**0/+**	**0/+**	**0**
**U**		11.5	12.0	13.5	10.5	12.0	13.0	44.0	29.0	31.0
***p***		0.006	0.008	0.011	0.005	0.008	0.011	0.970	0.235	0.302

Abbreviations: MSX2—muscle segment homeobox 2; PTX3—pentraxin 3; CISH—chromogenic in situ hybridization; Ep—epithelium; CT—connective tissue; End—endothelium; U—Mann–Whitney U test value; *p*—*p*-value; 0—no gene-signal-containing cells in the visual field; 0/+—a rare occurrence of gene-signal-containing cells in the visual field; +—a few gene-signal-containing cells in the visual field; +/++—few to moderate gene-signal-containing cells in the visual field; ++—moderate gene-signal-containing cells in the visual field; ++/+++—moderate to numerous gene-signal-containing cells in the visual field; +++—numerous gene-signal-containing cells in the visual field.

**Table 3:** Statistically significant correlations between factor-positive and/or gene-signal-containing cells in the control group.

Strength of Correlation	Correlation Between Factors	r_s_	*p*
Very strong positive (0.8–1.0)	*RYK* in Ep (CISH) and *MSX2* in End (CISH)	1.000	<0.001
	NFκB p65 in End (IHC) and NFκB p65 in CT (IHC)	0.984	<0.001
	*MSX2* in End (CISH) and *MSX2* in CT (CISH)	0.950	0.004
	*RYK* in Ep (CISH) and *MSX2* in CT (CISH)	0.950	0.004
	CCL4 in End (IHC) and NFκB p65 in End (IHC)	0.904	0.013
	CCL4 in CT (IHC) and NFκB p65 in CT (IHC)	0.898	0.015
	*MSX2* in CT (CISH) and *MSX2* in Ep (CISH)	0.890	0.018
	CCL4 in End (IHC) and NFκB p65 in CT (IHC)	0.889	0.018
	MSX1 in CT (IHC) and MSX1 in Ep (IHC)	0.874	0.023
	*MSX2* in End (CISH) and *MSX2* in Ep (CISH)	0.874	0.023
	*RYK* in Ep (CISH) and *MSX2* in Ep (CISH)	0.874	0.023
	*RYK* in End (CISH) and *MSX2* in End (CISH)	0.850	0.032
	*RYK* in End (CISH) and *RYK* in Ep (CISH)	0.850	0.032
	*PTX3* in End (CISH) and *PTX3* in Ep (CISH)	0.849	0.033
	*PTX3* in End (CISH) and *PTX3* in CT (CISH)	0.839	0.037
	CCL4 in CT (IHC) and NFκB p65 in End (IHC)	0.822	0.045
	CCL4 in CT (IHC) and RYK in CT (IHC)	0.822	0.045
Very strong negative (−1.0…−0.8)	*MSX2* in End (CISH) and NFκB p65 in CT (IHC)	−0.820	0.046
	*RYK* in Ep (CISH) and NFκB p65 in CT (IHC)	−0.820	0.046
	*RYK* in CT (CISH) and CCL4 in CT (IHC)	−0.822	0.045
	*MSX2* in Ep (CISH) and RYK in Ep (IHC)	−0.826	0.043
	*MSX2* in End (CISH) and CCL4 in End (IHC)	−0.839	0.037
	*RYK* in Ep (CISH) and CCL4 in End (IHC)	−0.839	0.037
	*MSX2* in CT (CISH) and RYK in CT (IHC)	−0.867	0.025
	*MSX2* in Ep (CISH) and RYK in CT (IHC)	−0.890	0.018
	*RYK* in CT (CISH) and CCL4 in End (IHC)	−0.904	0.013
	*RYK* in CT (CISH) and NFκB p65 in CT (IHC)	−0.984	<0.001
	*RYK* in CT (CISH) and NFκB p65 in End (IHC)	−1.000	<0.001

Abbreviations: Ep—epithelium; CT—connective tissue; End—endothelium; IHC—immunohistochemistry; CISH—chromogenic in situ hybridization; MSX1—muscle segment homeobox-1; RYK—receptor-like tyrosine kinase; NFκB p65—nuclear factor kappa-light-chain-enhancer of activated B cells protein 65; CCL4—C-C motif chemokine ligand 4; MSX2—muscle segment homeobox 2; PTX3—pentraxin 3; r_s_ = Spearman’s Rho value, *p*—*p*-value.

**Table 4:** Statistically significant correlations between factor-positive and/or gene-signal-containing cells in the patient group.

Strength of Correlation	Correlation Between Factors	r_s_	*p*
Very strong positive (0.8–1.0)	*RYK* in End (CISH) and *RYK* in CT (CISH)	0.901	<0.001
	*RYK* in CT (CISH) and *RYK* in Ep (CISH)	0.852	<0.001
	*RYK* in End (CISH) and *RYK* in Ep (CISH)	0.822	<0.001
	NFκB p65 in Ep (IHC) and RYK in Ep (IHC)	0.802	<0.001
Strong positive (0.6–0.8)	*PTX3* in End (CISH) and *PTX3* in Ep (CISH)	0.789	<0.001
	CCL4 in End (IHC) and CCL4 in Ep (IHC)	0.786	0.001
	*PTX3* in Ep (CISH) and RYK in End (IHC)	0.740	0.002
	CCL4 in End (IHC) and MSX1 in Ep (IHC)	0.722	0.002
	*PTX3* in End (CISH) and RYK in End (IHC)	0.709	0.003
	NFκB p65 in End (IHC) and MSX1 in CT (IHC)	0.704	0.003
	*PTX3* in End (CISH) and *PTX3* in CT (CISH)	0.682	0.005
	NFκB p65 in CT (IHC) and MSX1 in Ep (IHC)	0.642	0.010
	*PTX3* in Ep (CISH) and NFκB p65 in Ep (IHC)	0.613	0.015
Moderate positive (0.4–0.6)	*PTX3* in Ep (CISH) and RYK in CT (IHC)	0.584	0.022
	RYK in End (IHC) and RYK in CT (IHC)	0.578	0.024
	CCL4 in End (IHC) and NFκB p65 in CT (IHC)	0.547	0.035
	*PTX3* in CT (CISH) and *PTX3* in Ep (CISH)	0.546	0.035
	CCL4 in Ep (IHC) and MSX1 in Ep (IHC)	0.534	0.036
	*MSX2* in End (CISH) and *MSX2* in CT (CISH)	0.534	0.040
	NFκB p65 in CT (IHC) and MSX1 in CT (IHC)	0.530	0.042
	*MSX2* in CT (CISH) and *MSX2* in Ep (CISH)	0.527	0.044
	NFκB p65 in CT (IHC) and MSX1 in End (IHC)	0.519	0.048
Moderate negative (−0.6…−0.4)	*MSX2* in CT (CISH) and CCL4 in End (IHC)	−0.595	0.019

Abbreviations: Ep—epithelium; CT—connective tissue; End—endothelium; IHC—immunohistochemistry; CISH—chromogenic in situ hybridization; MSX1—muscle segment homeobox-1; RYK—receptor-like tyrosine kinase; NFκB p65—nuclear factor kappa-light-chain-enhancer of activated B cells protein 65; CCL4—C-C motif chemokine ligand 4; MSX2—muscle segment homeobox 2; PTX3—pentraxin 3; r_s_ = Spearman’s Rho value; *p*—*p*-value.

## Data Availability

The original contributions presented in this study are included in the article. Further inquiries can be directed to the corresponding author.

## Abbreviations

The following abbreviations are used in this manuscript:

MSX1	Muscle segment homeobox 1
MSX2	Muscle segment homeobox 2
RYK	Receptor-like tyrosine kinase
NFκB p65	Nuclear factor kappa-light-chain-enhancer of activated B cells protein 65
CCL4	C-C motif chemokine ligand 4
PTX3	Pentraxin 3
IHC	Immunohistochemistry
CISH	Chromogenic in situ hybridization
RSU	Rīga Stradiņš University
NFκB	Nuclear factor kappa-light-chain-enhancer of activated B cells
WNT	Wingless-related integration site
TNF-α	Tumor necrosis factor alpha
DIG	Digoxigenin
DNP	Dinitrophenol
AP	Alkaline phosphatase
HRP	Horseradish peroxidase
Ep	Epithelium
CT	Connective tissue
End	Endothelium
SPSS	Statistical Product and Service Solutions
U	Mann–Whitney U test value
*p*	*p*-value
